# TBK1 directs WIPI2 against *Salmonella*

**DOI:** 10.1080/15548627.2016.1235126

**Published:** 2016-10-18

**Authors:** Keith B. Boyle, Teresa L. M. Thurston, Felix Randow

**Affiliations:** a MRC Laboratory of Molecular Biology, Division of Protein and Nucleic Acid Chemistry, Francis Crick Avenue, Cambridge, UK; b MRC Center for Molecular Bacteriology and Infection, Imperial College, London, Flowers Building, Exhibition Road, London, UK; c University of Cambridge, Department of Medicine, Addenbrooke's Hospital, Cambridge, UK

**Keywords:** antibacterial autophagy, NDP52, optineurin, PI(3)P, *Salmonella*, TBK1, WIPI

## Abstract

Defense of the mammalian cell cytosol against *Salmonella* invasion is reliant upon capture of the infiltrating bacteria by macroautophagy (hereafter autophagy), a process controlled by the kinase TBK1. In our recent study we showed that recruitment of TBK1 activity to *Salmonella* stabilizes the key autophagy regulator WIPI2 on those bacteria, a novel and essential function for TBK1 in the control of the early steps of antibacterial autophagy. Substantial redundancy exists in the precise recruitment mechanism for TBK1 because engagement with any of several *Salmonella*-associated ‘eat-me’ signals, including host-derived glycans, and K48- and K63-linked ubiquitin chains, suffices to recruit TBK1 functionality. We therefore propose that buffering TBK1 recruitment against potential bacterial interference might be of evolutionary advantage to the host.

Antibacterial autophagy provides potent cell autonomous immunity against bacteria attempting to colonize the host cytosol. While professional cytosol-dwelling bacterial species, for example *Shigella flexneri*, have evolved countermeasures against autophagy, other opportunistic cytosolic invaders, such as *Salmonella enterica* serovar Typhimurium, fall prey to autophagic capture, which restricts their proliferation. Autophagy relies on the coordinated action of a suite of autophagy-related (ATG) proteins that control the initiation, elongation and closure of a phagophore to form an autophagosome around its cargo. The precise *in situ* initiation of antibacterial autophagy relies on the recognition of ‘eat-me’ signals associated with cytosol-exposed bacteria by specific cargo receptor proteins. The ‘eat-me’ signal LGALS8/Galectin-8 is recruited to sites of vacuolar membrane damage by exposure to the cytosol of otherwise hidden intralumenal glycans (Fig. 1). Ubiquitin chains deposited on bacteria and/or the damaged membrane remnants provide an independent ‘eat-me’ signal. The cargo receptor CALCOCO2/NDP52 recognizes both LGALS8 and ubiquitin chains whereas OPTN (optineurin) and SQSTM1/p62 bind the latter only. One crucial function of the cargo receptors is the tethering of the bacterial cargo to MAP1LC3/LC3 on the phagophore membrane. In addition to core autophagy genes, antibacterial autophagy also requires the TBK1 (TANK binding kinase 1) protein, whose autophagy-related function has remained incompletely understood.

**Figure 1.**
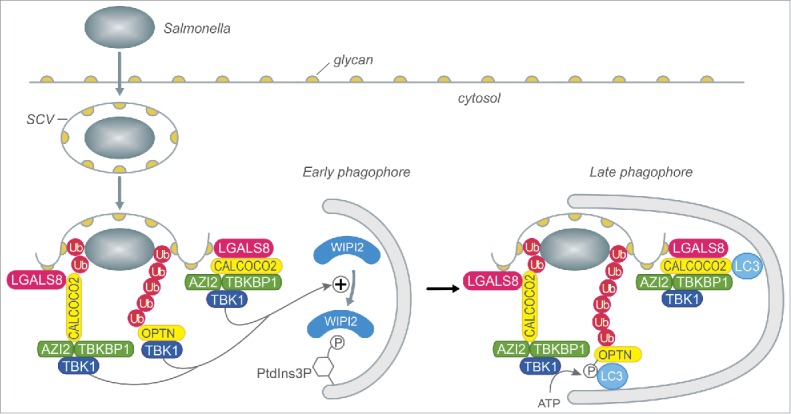
Upon invading host cells *Salmonella enterica* serovar Typhimurium resides in *Salmonella*-containing vacuoles (SCV), from which a minor fraction of bacteria gain access to the cytosol where autophagy restricts their otherwise excessive proliferation. Hitherto hidden glycosylated proteins and lipids (glycans) exposed on damaged SCVs recruit LGALS8, while host E3 ligases deposit poly-ubiquitin on the bacterium and/or damaged membranes, which together provide ‘eat-me’ signals for the cargo receptors CALCOCO2 and OPTN. CALCOCO2 recruits the kinase TBK1 via the adaptor proteins AZI2 or TBKBP1, whereas OPTN directly binds TBK1. Our new work reveals i) that recruitment of TBK1 via LGALS8 or ubiquitin chains of different linkage types is sufficient for antibacterial autophagy, and ii) that TBK1 enhances the recruitment of WIPI2 onto adjacent PtdIns3P-positive phagophores. Cargo receptors tether autophagy cargo to the phagophore, with TBK1-mediated phosphorylation of OPTN increasing its affinity for LC3. ATP, adenosine triphosphate; PtdIns3P, phosphatidylinositol 3-phosphate; SCV, *Salmonella-*containing vacuole; Ub, ubiquitin; LGALS8, galectin-8; AZI2, NAP1; TBKBP1, SINTBAD; CALCOCO2, NDP52.

TBK1 is a convergence point of multiple immune signaling pathways that originate at various toll-like receptors, RIG-I like receptors and TMEM173/STING. The canonical antiviral activity of TBK1 relies on phosphorylation of the transcription factor IRF3, a mode of action not required for its antibacterial effects. However, it is known that TBK1 localizes to cytosol-exposed *S.* Typhimurium and that it constitutively interacts with autophagy cargo receptors, either indirectly—via the adaptor proteins AZI2/NAP1 and TBKBP1/Sintbad—in the case of CALCOCO2, or directly in the case of OPTN. The phosphorylation of OPTN by TBK1 during antibacterial autophagy increases OPTN's affinity for LC3. Despite these findings it has remained enigmatic as to whether spatial control of TBK1 is important for antibacterial autophagy and, if so, which of the various recruitment mechanisms are important for its function.

In order to address this question we generated a deletion mutant of TBK1 (TBK1ΔC), which fails to bind any of its known adaptor proteins and is therefore not recruited to cytosol-invading *Salmonella*. We tested whether fusing TBK1ΔC directly to cargo receptors and ‘eat-me’ signals could rescue the antibacterial autophagy phenotype in TBK1-deficient MEFs. We found that antibacterial autophagy requires TBK1 kinase activity in the proximity of the bacterium and that considerable redundancy exists in the mechanism by which TBK1 is recruited. For example, fusion to either OPTN or CALCOCO2 rescues TBK1ΔC functionality as long as the cargo receptors retain binding sites for their cognate ‘eat-me’ signals. Moreover, TBK1ΔC function is rescued by directly fusing it to the ‘eat-me’ signal LGALS8 or to unrelated ubiquitin binding domains with specificity for K48 or K63 chains, thereby demonstrating that the recruitment of TBK1 to cytosol-invading bacteria is essential for antibacterial autophagy, while considerable functional flexibility exists regarding the precise recruitment signal. We therefore propose that promiscuity in the mechanism of TBK1 recruitment serves two purposes; i) it permits flexibility should bacteria become dissociated from any single ‘eat-me’ signal and ii) it acts as a buffer against potential bacterial interference with TBK1 recruitment.

While our study provided unambigious epistatic evidence that TBK1 exerts its antibacterial effects via autophagy, we made the striking observation that the recruitment of LC3 to cytosolic *Salmonella* is unaffected by loss of TBK1. A similar finding was reported previously for ATG9-deficient cells, which also retain apparently normal levels of LC3 recruitment to *Salmonella* while failing to restrict bacterial proliferation due to the conjugation of LC3 to the single vacuolar membrane rather than phagophores *sensu stricto.* Therefore, researchers must exercise caution when assigning recruitment of LC3 to bacteria as being a bona fide marker of conventional antibacterial autophagy.

Such a degree of flexibility in the recruitment of TBK1 begs the question of whether any means of directing TBK1 to cytosolic bacteria is sufficient. Apparently, not all bacteria-associated potential ligands are equal because the LC3-interacting regions of OPTN and CALCOCO2 are insufficient to rescue the function of TBK1ΔC, suggesting that LC3 lipidation to the forming phagophore lies downstream of a hitherto unidentified function of TBK1. We therefore investigated whether the recruitment of upstream autophagy proteins to cytosolic *S.* Tymphimurium was dependent on TBK1. Indeed, we found that the recruitment of WIPI2, a PtdIns3P and PtdIns(3,5)P_2_ binding protein itself essential for antibacterial autophagy, requires TBK1 activity in the proximity of cytosol-invading bacteria (Fig. 1). Intriguingly, TBK1 does not influence the synthesis of PtdIns3P or PtdIns(3,5)P_2_, as recruitment of fluorescent probes for these lipids to bacteria is unaffected by loss of TBK1. Thus we propose that WIPI2 acts as a coincidence detector of both TBK1 and PIK3C3/VPS34 activity, thereby ensuring specific recruitment of WIPI2 to cytosolic bacteria.

The obvious question at this point is the identity of the TBK1 substrate. Is it WIPI2 itself or some other unknown protein? For now it seems clear that functionally important TBK1 substrates other than OPTN must exist because cells lacking OPTN retain recruitment of WIPI2. It remains to be tested whether the proposed phosphorylation of CALCOCO2 or SQSTM1 by TBK1 is important in this context, but it is clear that identification of TBK1 substrate(s) will form the basis of future research efforts.

